# Laser-Driven Modulation of Electron Beams in a Dielectric Micro-Structure for X-Ray Free-Electron Lasers

**DOI:** 10.1038/s41598-019-56201-8

**Published:** 2019-12-24

**Authors:** Benedikt Hermann, Simona Bettoni, Thilo Egenolf, Uwe Niedermayer, Eduard Prat, Rasmus Ischebeck

**Affiliations:** 1 Paul Scherrer Institut, 5232 Villigen PSI, Switzerland; 2 Institute of Applied Physics, University of Bern, 3012 Bern, Switzerland; 3 Institute for Accelerator Science and Electromagnetic Fields (TEMF), TU Darmstadt, 64289 Darmstadt, Germany

**Keywords:** Optics and photonics, Applied optics, Lasers, LEDs and light sources, Optical materials and structures

## Abstract

We describe an application of laser-driven modulation in a dielectric micro-structure for the electron beam in a free-electron laser (FEL). The energy modulation is transferred into longitudinal bunching via compression in a magnetic chicane before entering the undulator section of the FEL. The bunched electron beam comprises a series of enhanced current spikes separated by the wavelength of the modulating laser. For beam parameters of SwissFEL at a total bunch charge of 30 pC, the individual spikes are expected to be as short as 140 as (FWHM) with peak currents exceeding 4 kA. The proposed modulation scheme requires the electron beam to be focused into the micrometer scale aperture of the dielectric structure, which imposes strict emittance and charge limitations, but, due to the small interaction region, the scheme is expected to require ten times less laser power as compared to laser modulation in a wiggler magnet, which is the conventional approach to create a pulse train in FELs.

## Introduction

Free-electron lasers (FELs) make use of a highly compressed relativistic electron beam to generate electromagnetic radiation in a magnetic undulator. They are the brightest sources of radiation from the VUV^1,2^ to the X-ray regime^3–7^. The emission of radiation in a so-called self-amplification of spontaneous emission (SASE) FEL grows exponentially from noise and critically depends on the local properties of the electron beam. A technique proposed to generate an individual or a train of sub-femtosecond X-ray pulses with well-defined separation is the energy modulation of the electron bunch with a laser in the magnetic field of a resonant undulator. This “enhanced SASE” (ESASE) method has been proposed by Zholents^8^, and is being implemented at LCLS^9^ and planned at other facilities such as SwissFEL^10^.

**Figure 1:**
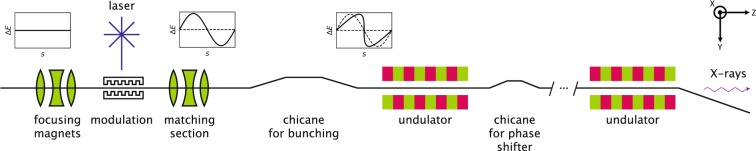
Schematic overview of the proposed scheme. Strong focusing is required to match the beam into the micrometer scale aperture of the dielectric grating. The longitudinal phase-space is modulated by the optical near fields in the dielectric structure excited by a laser. The energy modulation is converted into longitudinal bunching by a magnetic chicane. Three inset plots illustrate the longitudinal phase-space evolution. The resulting pulse train emits a series of homogeneously spaced x-ray pulses which can be mode-locked by small chicanes between undulator modules acting as phase shifters.

In this paper, we present and compare an alternative method to the conventional undulator modulation scheme. We show, that by modulating the electron bunch in a laser-driven dielectric micro-structure, similar modulation strength can be achieved with significantly lower laser power. The beamline required for the proposed scheme is sketched in Fig. 1. Key elements are the strong focusing and matching quadrupole triplets, the chicane for bunching and the FEL undulator section with interleaved chicanes acting as phase shifters. The intrinsic synchronization of the FEL pulses to an external laser, which is achieved in both schemes, naturally gives rise to pump-probe experiments. The CHIC scheme in the soft X-ray beamline in SwissFEL (Athos) makes use of magnetic chicanes between the undulator segments to delay the electron beam with respect to the X-Rays^10,11^. Athos is planned to deliver FEL radiation for wavelengths ranging from 0.65 nm to 5 nm from 2020 and user operation starting in 2021^10^. Adjusting these chicanes to form overlap between each X-ray pulse with the subsequent slice of the electron bunch, the longitudinal coherence is transferred along the bunch, and the X-ray pulses become phase-locked^12^. The chamber for the interaction with the laser and dielectric structure is installed in the switchyard to Athos and is currently being commissioned. It is also planned to demonstrate GV/m gradients in these dielectric laser acceleration (DLA) structures with a length of 1 mm at a laser wavelength of 2 μm^13,14^.

## Modulation of Electron Beams in a Laser-Driven Dielectric Double Grating

Illuminating a dielectric double grating with a laser creates evanescent waves which travel inside the gap of the structure with a phase velocity defined by the periodicity of the structure and the wavelength of the illuminating laser. For a laser incident normal onto a straight grating a net interaction with a charged particle traveling along the gap is achieved if the resonance condition is fulfilled: ${\lambda }_{S}={\lambda }_{L}\beta n$^15^. Here, *λ*_*S*_ denotes the periodicity of the dielectric structure, *λ*_*L*_ is the wavelength of the laser, *β* is the normalized electron velocity and *n* is the order of the spatial harmonic of the evanescent wave. To sustain the interaction over distances greater than the laser pulse length, a pulse-front tilt setup for the laser has to be employed^16–18^. Due to the optical phase dependence of the interaction, net-acceleration of electrons can be achieved only for bunches significantly shorter than the wavelength of the driving laser. The momentum distribution of an electron beam longer than the laser wavelength will be modulated sinusoidally. The amplitude of the longitudinal interaction with the evanescent waves inside the channel of a straight grating is proportional to $\cosh (2\pi y/({\lambda }_{L}\beta \gamma ))$ and the transverse component of the interaction is proportional to $\sinh (2\pi y/({\lambda }_{L}\beta \gamma ))$^19^. As a result, the modulation amplitude becomes homogeneous over the entire gap and the transverse component vanishes for ultra-relativistic particles ($\gamma \gg 1$). Therefore, a straight grating illuminated by a laser polarized along the direction of the electrons creates an almost purely longitudinal momentum modulation. A resonant transverse momentum component can be added by tilting the grating^20^. In principle, the transverse momentum modulation can be used to bunch the beam by an appropriate compression setup using *R*_52_ (transfer matrix element relating transverse momentum changes to temporal deviations). However, compression of the transversely modulated beam leads to a slice emittance increase within the spikes. Since the SASE process strongly depends on the transverse slice emittance^21^, we focus on longitudinal modulation only to avoid emittance growth after compression.

## Results

### Modulation and compression

**Figure 2:**
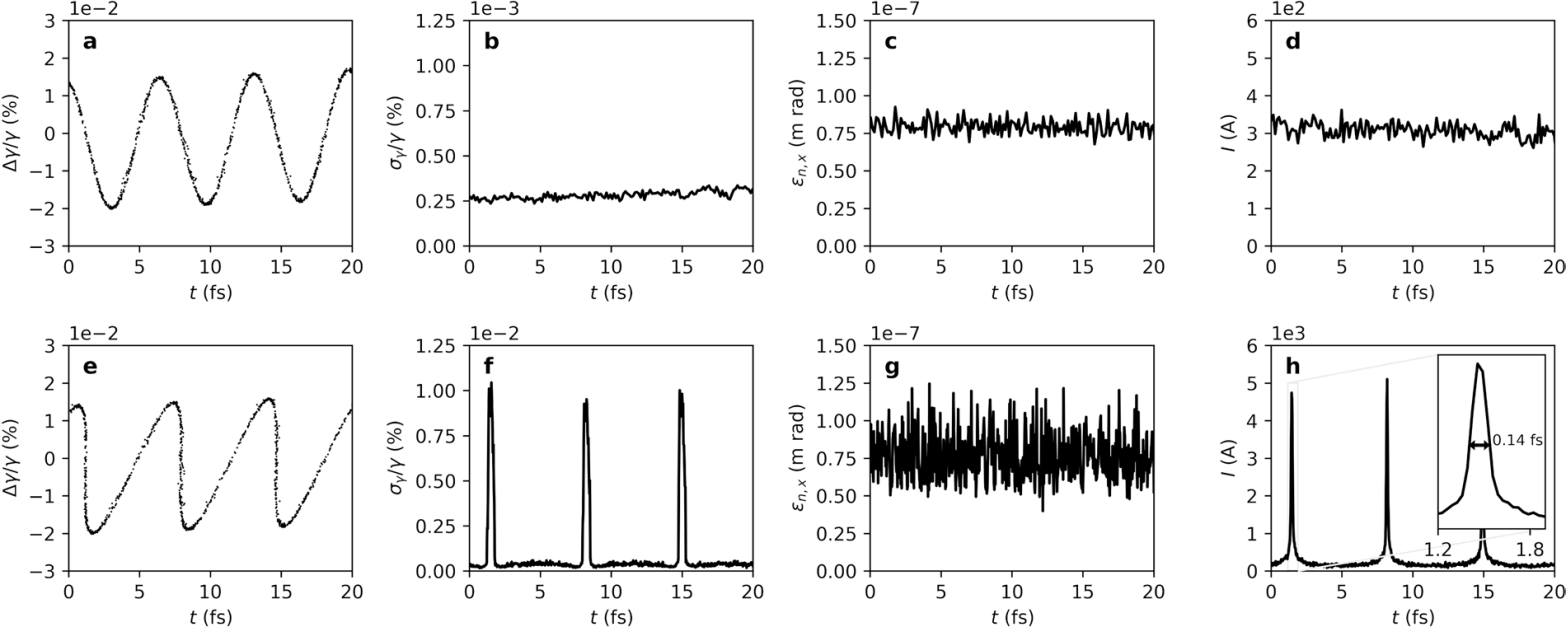
Simulated phase-space of the 3 GeV electron beam of SwissFEL at two locations: after the DLA interaction (first row, (**a**–**d**)) and after the propagation through the switchyard to the Athos beamline and optimal compression (second row, (**e**–**h**)) A strong increase in slice energy spread is observed, compare sub-figure (**b** and **f**), whereas the slice emittance remains unchanged (sub-figure (**c** and **g**)). The noise in (**g**) appears larger than in (**c**), as the bin width is reduced to resolve the short spikes. After optimal compression peak currents of up to 5 kA are predicted by this simulation (**h**).

The particle distribution for this study is optimized with ASTRA^22^ to obtain a low emittance and a low energy spread for a bunch charge of 30 pC and an energy of 3 GeV to achieve a final peak current which is sufficient to drive the SASE FEL process. This working point is covered by the parameter range of the SwissFEL accelerator at the location of the ACHIP interaction chamber in the switchyard of the Athos beamline. At this location, the optimized distribution has a peak current of 300 A and a length of around 100 fs. The tracking of the modulated electron beam through the switchyard is done with ELEGANT^23^ including longitudinal space charge and coherent synchrotron radiation effects. The electromagnetic field distribution created in a laser excited structure is modeled with CST Studio^24^. We observe that the longitudinal and transverse component of the interaction follow the analytical solution derived in^19^. The remaining transverse modulation amplitude is 500 times smaller than the transverse momentum spread for the case of the 3 GeV electron beam in Athos at SwissFEL. Detailed information about the field simulations with CST are given in section Methods -. We conclude, that the interaction of an ultra-relativistic electron bunch ($\gamma \approx 6000$) inside the channel of a double grating structure with the fields of a normally incident laser can be modeled as a homogeneous sinusoidal momentum modulation. The transverse kicks and the transverse dependence of the longitudinal kick vanish for a straight grating and ultra-relativistic electrons^19^. The symmetry of the field can be even further enhance by illuminating the structure from both sides or by using a distributed Bragg mirror (DBR) behind the structure^25,26^. The DBR consists of dielectric layers with well-defined thicknesses to reflect the laser and mimic double-sided illumination. The modulated electron beam is bunched in a subsequent magnetic chicane. Naturally, the achievable peak current after optimal compression depends on the slice energy spread and increases with the modulation strength, up to a certain limit. To illustrate the concept we use a modulation amplitude of 0.5 MeV, which leads to an optimal *R*_56_ around 2 mm. This modulation amplitude corresponds to an average acceleration gradient of 0.5 GV/m in a 1 mm long structure. Such gradients have recently been demonstrated in a DLA experiment for relativistic electrons at the Pegasus facility at UCLA in 0.5 mm to 1 mm long structures^27^. The interaction length was limited to 21.5 μm due to the temporal overlap with the laser pulse since no pulse-front tilt for the laser was used in this experiment. The simulation results for the DLA modulation, transport through the Athos switchyard of SwissFEL and optimal bunching are summarized in Fig. 2. In the first row (sub-figures a–d) projections of the phase-space just after the DLA interaction are shown, whereas the second row (sub-figures e–h) depicts the bunched particle distribution after the magnetic chicane. After compression a strong increase of the slice energy spread (${\sigma }_{\gamma }/\gamma =1\times {10}^{-4}$) within the current spikes is observed, where *γ* is the relativistic mass factor. For SASE FELs the energy spread has to be much smaller than the FEL Pierce parameter *ρ*, see for instance^28^. In case of the Athos beamline at SwissFEL, *ρ* is in the order of 1 × 10^−3^, this condition is fulfilled. Since the transverse modulation is negligible, we assume the slice emittance (${\varepsilon }_{n,x}$) to be conserved. Peak currents of up to 5 kA are achievable in this configuration, which corresponds to a current enhancement by a factor of 16. Similarly as for the conventional scheme, the spike heights inherit the Gaussian envelope from the original macro-bunch shape. The length of the individual spikes is expected to be around 140 as (FWHM). We observe 14 individual spikes with peak currents exceeding 2.5 kA spaced by 6.7 fs corresponding to the laser wavelength of 2 μm.

### Comparison with undulator modulation

The conventional ESASE scheme uses an undulator magnet to resonantly transfer energy from a co-propagating optical laser to the electron beam to achieve a net-modulation of the electron beam^8^. Here, we compare our proposed DLA modulation scheme to the conventional approach in terms of electron beam and laser requirements.

#### Emittance and charge limitations

**Figure 3:**
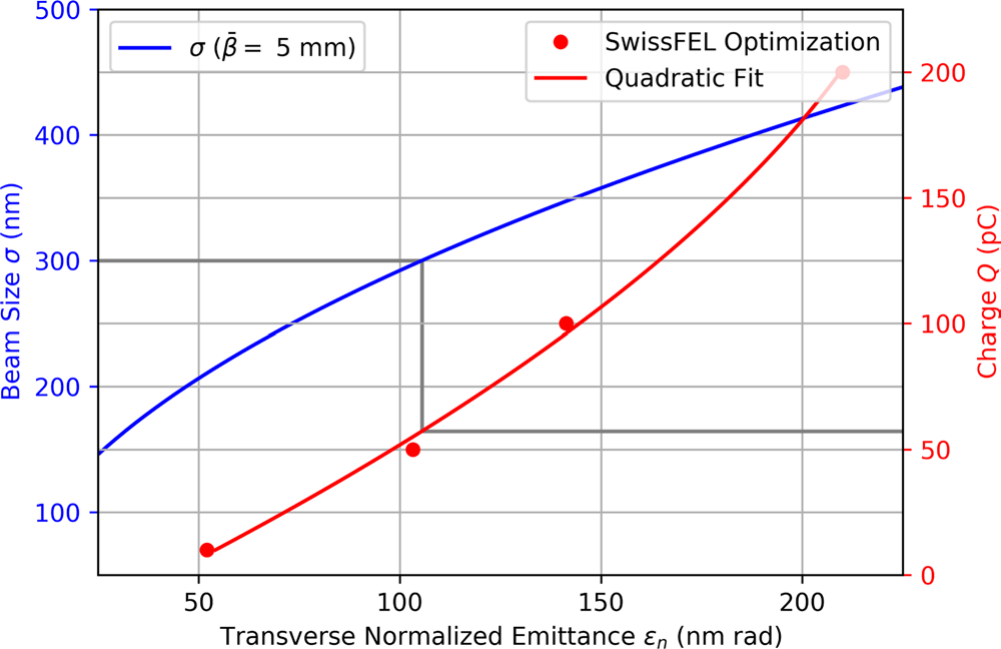
The transverse beam size (*σ*) for a fixed $\bar{\beta }$-function (5 mm) and the maximum charge are plotted as a function of emittance. The emittance has been optimized for the SwissFEL injector at 4 different working points. We observe that a maximum charge of 50 pC can be focused to a beam size of 0.3 μm using the existing magnets in the ACHIP chamber installed in the Athos switchyard of SwissFEL.

Typical apertures of wiggler magnets are in the order of 10 mm. Typical electron beam sizes at the end of FEL accelerators are far below 1 mm. Thus, no special focusing elements for the electron beam are needed and the charge which can be transported through the modulating structure is not limited by the geometry of the wiggler. In comparison, the DLA modulation scheme requires the GeV electron beam to be focused into the micrometer scale channel of the dielectric structure. This can be achieved with strong quadrupole focusing in combination with low transverse emittance. The beam size is determined by the emittance and the Twiss parameter $\bar{\beta }$ at the interaction point by $\sigma ={({\varepsilon }_{n}\bar{\beta }/\gamma )}^{1/2}$. Since emittance scales with the charge for photo-injectors used at FELs, the charge which can be modulated in this scheme is limited. To estimate the charge limit, we assume a grating aperture of 1.2 μm, a beam energy of 3 GeV, and a $\bar{\beta }$-function of 5 mm at the interaction point. These parameters can be achieved with the existing permanent magnet quadrupoles in the ACHIP chamber in the Athos branch at SwissFEL^14^. The in-vacuum quadrupole magnets are 10 cm long and provide a geometric strength (*K*-value) of 26 m^−2^ and 39 m^−2^ at a beam energy of 3 GeV^13^. For the parameters described above, the beam size and charge are plotted against emittance in Fig. 3. The emittance of the electron beam in SwissFEL has been optimized for different charges according to the procedure explained in Methods. For a maximum beam size of *σ* = 0.3 μm, corresponding to a 4*σ*-aperture of 1.2 μm, a normalized emittance smaller than 100 nm rad is required, which limits the charge to approximately 50 pC.

#### Modulation effectiveness

The total modulation amplitude Δ*γ* obtained from a laser with a peak power *P*_*pk*_ and a spot size *σ*_*L*,*x*_, *σ*_*L*,*z*_ (distance from the center at which the intensity drops to *e*^−1/2^ of the maximum), in a dielectric grating of length *L*_*S*_, is calculated by

1 $$\Delta \gamma ={e}_{1}\frac{e}{{m}_{e}{c}^{2}}{(\frac{{P}_{pk}}{c{\varepsilon }_{0}\pi {\sigma }_{L,x}{\sigma }_{L,z}})}^{\frac{1}{2}}\,{\int }_{-{L}_{S}/2}^{{L}_{S}/2}\,\exp \,(\,-\frac{{z}^{2}}{4{\sigma }_{L,z}^{2}})\,{\rm{d}}z.$$

Here, *e*_1_ denotes the structure factor being the ratio of the effective acceleration gradient to the required incident electric field strength, which corresponds to the Fourier coefficient of the first spatial harmonic of the laser field inside the structure^19^. This formula is derived by integration of the electric field along the structure, and from the relation between the electric field vector $\overrightarrow{E}$ and the intensity *I* of an electromagnetic wave, $I=c{\varepsilon }_{0}\vec{{E}^{2}}/2$, where the vacuum permittivity is denoted by ${\varepsilon }_{0}$. Considering that the modulation amplitude is proportional to the square root of the laser power, it is useful to define the unit-less modulation effectiveness $\eta$ as

2 $$\eta =\frac{c{\varepsilon }_{0}}{{e}^{2}}\frac{{({m}_{e}{c}^{2}\Delta \gamma )}^{2}}{{P}_{pk}}.$$

**Figure 4:**
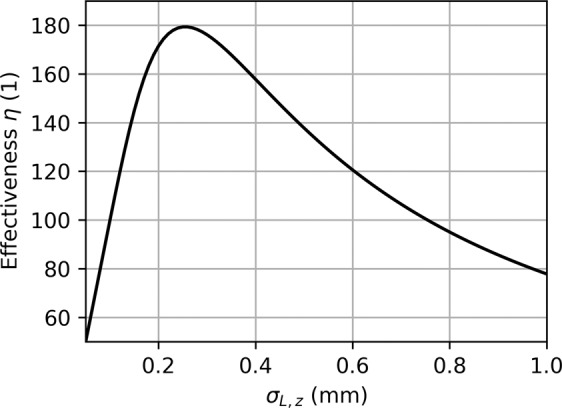
Dependency of the DLA modulation effectiveness on the laser focal spot size *σ*_*L*,*z*_ for a 1 mm long structure.

This quantity describes the effectiveness of the modulating process and can be applied to any laser driven modulation scheme. It is a measure for the laser power which is required to achieve a certain modulation amplitude. The effectiveness depends on the laser focal spot size as described by Eqs. 1 and 2. Due to the Gaussian integral involving the shape of the laser in *z*, the dependence is not monotonous and an optimum value can be found. To illustrate this, we plot the effectiveness against ${\sigma }_{L,z}$, see Fig. 4. For a 1 mm long structure, the maximum effectiveness is achieved for ${\sigma }_{L,z}\approx 250$ μm. In practice, it can be favorable to use a larger laser spot size to reduce the peak electric field strength in the dielectric material and accept the reduced effectiveness. For the comparison, we assume a laser focal spot size of ${\sigma }_{L,x}=4$ μm by ${\sigma }_{L,z}=500$ μm. The effectiveness is approximately 20% lower as compared to the optimal case, see Fig. 4. The Rayleigh length in *x* for a Gaussian laser beam is ${z}_{r,x}=4\pi {\sigma }_{L,x}^{2}/{\lambda }_{L}$, which is around 100 μm in this case. The structure factor *e*_1_ for a relativistic DLA made of fused silica is typically in the order of 1, which is what we use for the calculation, but can be higher (2–3) for a smaller gap size^29^. Higher *e*_1_ however goes along with more resonant structures, prohibiting short pulse operation. Based on these parameters and the formula above we calculate an effectiveness of the DLA modulation technique of around 140. The ESASE scheme proposed for LCLS at Stanford uses a modulation at a wavelength of 2.2 μm with a modulation amplitude of 7 MeV. The required laser peak power is estimated to be 10.7 GW^30^. The corresponding effectiveness, as previously defined, for this setup is around 12. In comparison, the proposed DLA modulation scheme is around ten times more effective, in terms of required laser power for a fixed modulation amplitude. The requirements for the electron beam and the laser pulse for both schemes are summarized in Table 1.

**Table 1:** Comparison of the requirements for the electron beam and the laser for DLA modulation and conventional undulator modulation for ESASE.

Parameter	Symbol	DLA Modulation	ESASE Undulator	Unit
Required transverse electron beam size	*σ*_*e*,*y*_	0.3	—	μm
Structure Period (grating/wiggler magnet)	*λ*_*S*_	2 × 10^−6^	0.3	m
Number of periods	*N*_*S*_	500	8	1
Structure length	*L*_*S*_	1 × 10^−3^	2.4	m
Laser wavelength	*λ*_*L*_	2	2.2	μm
Laser spot size, x	*σ*_*L*,*x*_	4	250	μm
Laser spot size, z	*σ*_*L*,*z*_	500	250	μm
Modulation effectiveness	*η*	140	12	1

The parameters for the ESASE scheme are taken from the proposal for LCLS^30^.

#### Tunability

**Figure 5:**
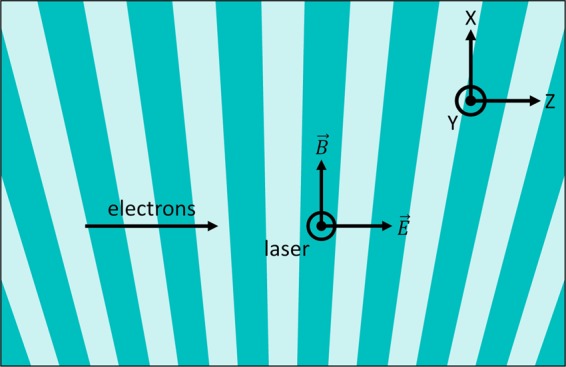
Conceptual drawing of the diverging grating structure. The periodicity can be scanned by moving the grating along *x* with respect to the electron beam. The dimensions and especially the slope of the changing periodicity need to be chosen, such that the periodicity changes not significantly within the beam size. As a consequence, the length of the chirped grating along the open direction (*x*) follows from the required tunability range. For a homogeneous interaction within the transverse beam shape we limit the periodicity change within 10 μm to $0.01{\lambda }_{S}\equiv 20\,{\rm{nm}}$. The resulting slope of the periodicity change along *x* equals 2 × 10^−3^. For a tunability range of 1.5 μm to 2.5 μm a structure length along the open direction (*x*) is given by wavelength range/slope = 0.5 mm.

Another important aspect for both schemes is the tunability of the modulation period. In both scenarios a change of the laser wavelength *λ*_*L*_ is required. In an experiment, this can be realized by a tunable optical parametric amplifier (OPA) in combination with reflective optics for the laser transport to avoid chromatic effects, for example in lenses. Regarding the undulator modulation scheme, the wiggler parameter *K*_*W*_ needs to be modified such that ${\lambda }_{L}={\lambda }_{W}(1+{K}_{W}^{2}/2)/2{\gamma }^{2}$, where *λ*_*W*_ is the wiggler period^8^. This is typically achieved by adjusting the gap between the magnetic arrays of the wiggler. In order to change the modulation period in the DLA scheme, the periodicity of the structure needs to be modified according to the resonance condition ${\lambda }_{S}={\lambda }_{L}\beta n$. We propose to realize this with a chirped (diverging) grating, where the periodicity slowly changes along the open direction, see Fig. 5. The resulting tilt of the grating will not degrade the transverse emittance since the transverse momentum modulation acquired in the first half of the structure is canceled in the second half as the tilt angle is inverted. In comparison to a series of different structures, the chirped grating approach would provide fast and continuous scanning capabilities by positioning the grating along its open direction (x). In principle, fused silica can be used for wavelengths ranging from 0.4 μm to 4 μm. In this window, the refractive index at room temperature varies between 1.4 and 1.6 and the absorption index is close to zero. To enhance the modulation effectiveness other grating parameters, such as the grating teeth width, could be optimized for the desired wavelength interval. For larger wavelengths, the absorption rises strongly and peaks at around 10 μm^31^. The length of the individual current spikes can be tuned in both schemes by adjusting the modulation amplitude and the compression factor *R*_56_ of the magnetic chicane. For the DLA modulation scheme the modulation amplitude is limited by the damage threshold of the dielectric structure. Irreversible damage of fused silica DLAs has been observed for incident electric fields of around 9 GV/m^27^, which would limit the modulation amplitude to around 9 MeV for a 1 mm long structure. This boundary is more than one order of magnitude higher than the modulation amplitude we use for the bunching simulation presented above.

## Discussion

We investigated a scheme which uses the fields in a dielectric micro-structure excited by a laser to modulate the ultra-relativistic electron beam of an FEL with the purpose of creating a pulse train for the ESASE scheme. Beam dynamics simulations using parameters of the SwissFEL accelerator predict the generation of 140 as (FWHM) electron spikes with peak currents up to 5 kA from a bunch with a total charge of 30 pC and an initial current of 0.3 kA. These beam parameters are suitable to drive the SASE process and create pulsed FEL radiation. Investigation of the properties of the radiation created in the Athos undulators at SwissFEL, through time-dependent FEL simulations, can be subject of a future study. Especially, the undulator tapering to increase the pulse energy while keeping the FEL pulses short and phase shifters between the undulator sections to create phase-locked pulses need to be optimized in detail.

The micrometer scale aperture of the dielectric structure implies strict limitations for beam focusing and transverse emittance. For the case of the Athos beamline at SwissFEL, the estimated charge limit is around 50 pC. Losses created by the tails of the beam limit the achievable repetition rate. The conventional undulator modulation scheme has no such limitations and is favourable in a high charge, emittance and repetition rate machine. Based on our estimation, dielectric laser modulation for ultra-relativistic electron beams is around a factor of ten more effective than undulator modulation, meaning that only a tenth of laser power is required to achieve the same modulation amplitude of the longitudinal phase-space. Since the laser system is one of the main cost drivers of the ESASE scheme, the proposed DLA modulation scheme presents a significant economic advantage. This aspect is particularly important for future, more compact and less expensive X-ray FEL facilities which will operate at lower charge and emittance than existing facilities. Both schemes allow continuous scanning of the modulation period, by changing the wavelength of the laser and properties of the modulating structure: in the undulator modulation scheme the wiggler parameter needs to be changed; in the DLA modulation scheme the periodicity of the grating needs to be adapted. We propose to use a diverging grating for continuous scanning capabilities.

## Methods

### Emittance optimization for swissFEL

SwissFEL can run at different bunch charges to accommodate the requests of the users. For each case, the emittance and the optics mismatch along the slices of the beam have been optimized at the injector to maximize the uniformity of the properties along the bunch, and therefore the lasing intensity. The main parameters included in the optimization are the first focusing at the exit of the radio-frequency gun, along the first accelerating structure, and the transverse size of the laser at the cathode. These parameters are determined starting from the layout corresponding to the 200 pC design case of SwissFEL and finely tuned using a simplex optimizer^32^. In the simulations the assumed intrinsic emittance is 550 nm/mm, accordingly to what was measured at the SwissFEL injector test facility^33,34^. Downstream of the injector the beam is compressed in two stages. We optimized the compression parameters to have an optimum balance between the intrinsic energy spread, the peak current and the residual chirp. We simulated 2 × 10^5^ macro-particles for the tracking with ELEGANT^23^ of the 30 pC-distribution which is used in this study.

### Electron optics for high current beams

**Figure 6:**
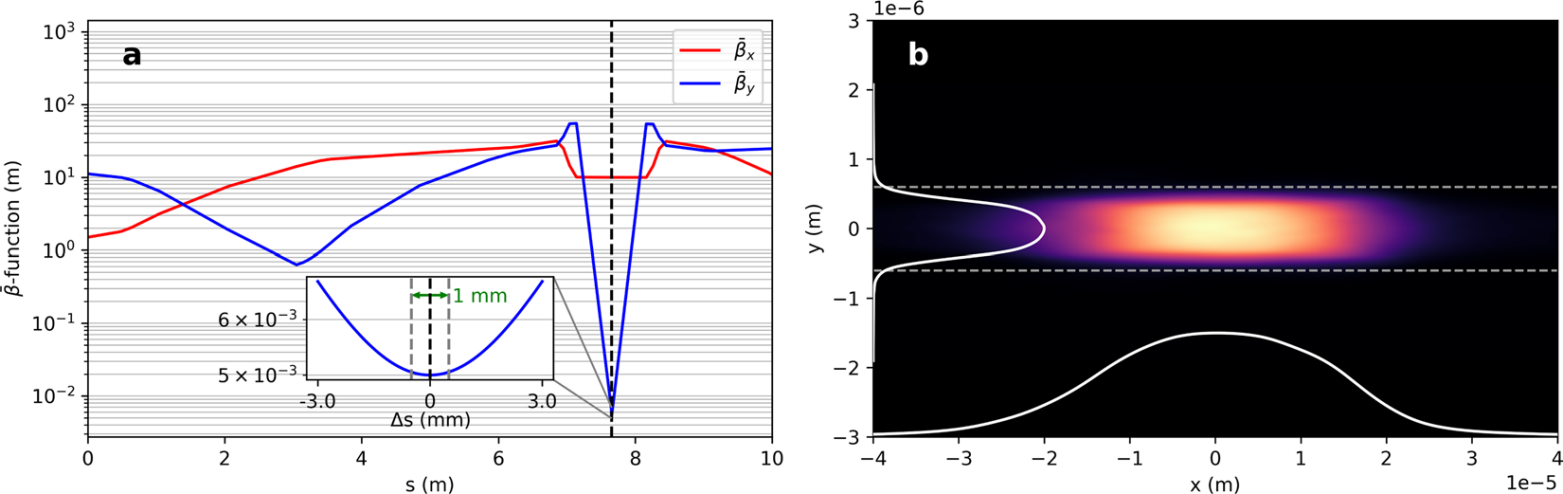
(**a**) Electron optics for asymmetric focusing with 2 strong permanent magnet quadrupoles. The Twiss parameters ${\bar{\beta }}_{x}$ and ${\bar{\beta }}_{y}$ are plotted around the interaction region. A ratio ${\bar{\beta }}_{x}/{\bar{\beta }}_{y}$ of around 1600 is achieved with a permanent magnet quadrupole doublet. The inlet shows the evolution of ${\bar{\beta }}_{y}$ in the vicinity of the interaction point (black dashed line). We observe that the beam size does not change significantly within a propagation distance of 1 mm (green arrow). (**b**) Simulated electron beam profile at the interaction point. The two dashed lines indicate the gap of the dielectric double grating (1.2 μm).

Operating the proposed DLA modulation scheme with a high current initial electron beam will induce strong wakefields inside the dielectric structure that may lead to heating and destruction of the device. The electron optics can be adapted to achieve a strongly asymmetric focus to reduce the current density inside the structure to reduce short-range wakefields. This can be achieved with a strong quadrupole doublet. We used the focusing strengths of the existing permanent magnets inside the ACHIP chamber at SwissFEL and optimized their position with ELEGANT^23^. Due to the ultra-relativistic energy (3 GeV) transverse space charge effects at the focus can be neglected and the particle tracking code ELEGANT can be applied. The laminarity parameter, as defined for instance in^35^, must not exceed unity in order to neglect space charge forces. For the parameters of this study the laminarity parameter at the interaction point is indeed around 1 × 10^−3^. The Twiss parameters (${\bar{\beta }}_{x}$ and ${\bar{\beta }}_{y}$) along the beamline and the transverse beam profile at the interaction point for a normalized slice emittance at the core the beam of 80 nm rad are shown in Fig. 6. The particle distribution for this simulation was optimized with ASTRA^22^ at a charge of 30 pC for the SwissFEL injector. The compression settings were optimized using ELEGANT. With the optimized permanent magnet configuration a ratio ${\bar{\beta }}_{x}/{\bar{\beta }}_{y}$ of around 1600 can be achieved (${\bar{\beta }}_{x}=10\,{\rm{m}}$, ${\bar{\beta }}_{y}=5\,{\rm{mm}}$). The aspect ratio of the electron beam is given by ${({\bar{\beta }}_{x}/{\bar{\beta }}_{y})}^{1/2}\approx 40$. A larger transverse electron beam size at the interaction point requires an even larger laser spot to maintain a homogeneous interaction. As a result, the laser power requirement would increase.

### Structure optimization and transverse effects

**Figure 7:**
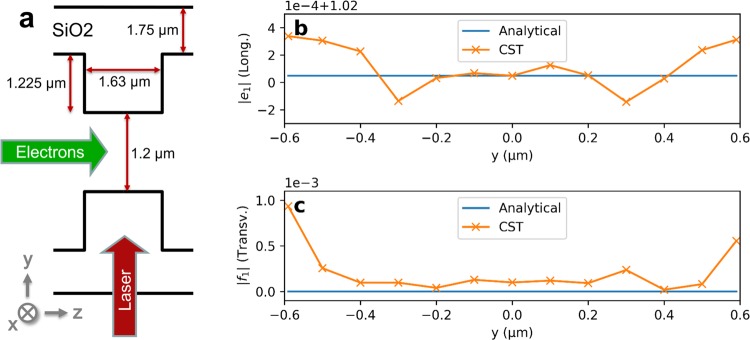
(**a**) Parameters of the optimized double grating. (**b**) Longitudinal structure factor *e*_1_ plotted along the 1.2 μm wide gap of the structure. The numerical noise of the constant structure factor of 1.02 is in the order of 1 × 10^−4^. (**c**) Transverse structure factor *f*_1_. Transverse forces (including numerical noise) across the gap are 3 orders of magnitude smaller than longitudinal forces.

The geometry of the dielectric double grating is optimized to achieve a structure factor of about 1. We used CST Microwave Studio^24^ to calculate the electromagnetic fields of a single grating period in the frequency domain. The incident laser field is modeled as a plane wave coupled into the dielectric structure. The Fourier coefficient of the first spatial harmonic is given by^19^

3 $${\underline{e}}_{1}(y)=\frac{1}{{\lambda }_{S}{E}_{0}}\,{\int }_{-{\lambda }_{S}/2}^{{\lambda }_{S}/2}\,{\underline{E}}_{z}(y,z)\,\exp \,(i\frac{2\pi }{{\lambda }_{S}}z)\,{\rm{d}}z$$

with the longitudinal electric field ${\underline{E}}_{z}(y,z)$ inside the channel and the amplitude *E*_0_ of the incident laser field. The absolute value of the complex Fourier coefficient as the structure factor describes the ratio of the acceleration gradient to the incident laser field. To achieve the desired structure factor in the center of the channel, we optimized the teeth and also the base thickness for a given aperture. The resulting parameters are shown in Fig. 7. Since the transverse dependence of the Fourier coefficient is analytically given as ${\underline{e}}_{1}(y)={\underline{e}}_{1}(y=0)\,\cosh \,(2\pi y/({\lambda }_{L}\beta \gamma ))$^19^, the modulation amplitude is almost independent of the transverse position in the gap. This is confirmed by numerically evaluating the structure factor along the gap, see Fig. 7. The transverse fields can be analytically obtained using the Panofsy-Wenzel theorem. For a straight grating, the transverse kick becomes ${\underline{f}}_{1}(y)={\lambda }_{L}/(2\pi )\partial {\underline{e}}_{1}/\partial y={\underline{e}}_{1}(y=0)\,\sinh \,(2\pi y/({\lambda }_{L}\beta \gamma ))/\gamma$^19^ and therefore vanishes for ultra-relativistic electrons. Numerically the transverse kick can be calculated by integrating the transverse electric and magnetic fields inside the channel as

4 $${\underline{f}}_{1}(y)=\frac{1}{{\lambda }_{S}{E}_{0}}\,{\int }_{-{\lambda }_{S}/2}^{{\lambda }_{S}/2}\,({\underline{E}}_{y}(y,z)+\beta c{\underline{B}}_{x}(y,z))\,\exp \,(i\frac{2\pi }{{\lambda }_{S}}z)\,{\rm{d}}z.$$

The evaluation of this integral along the gap using the fields simulated by CST Microwave Studio confirmed the analytical description, i.e. that the transverse effect vanishes, see Fig. 7. Note that the numerical noise arises especially from the interpolation of the magnetic field, which is not allocated at the same mesh entity as the electric field and thus needs to be interpolated. This is the reason why the numerical transverse kicks, which are close to zero, do not fulfill the Panofsky-Wenzel theorem. To estimate the effect of the remaining transverse component we compare *f*_1_ to the transverse momentum spread of the electron beam at the interaction point. Over the entire gap the transverse amplitude is more than a factor of 1000 smaller than the longitudinal modulation component. For the case of the longitudinal modulation amplitude used in this study (0.5 MeV) the transverse modulation is expected to be smaller than 0.5 keV. The rms transverse momentum spread at the interaction point is calculated to be around 260 keV, which is a factor of 500 larger than the transverse modulation effect. The large transverse momentum spread is a result of the strong focusing. Hence, the degradation of the slice emittance by transverse forces in the structure can be neglected for an ultra-relativistic electron beam.

### Jitter and stability considerations

**Figure 8:**
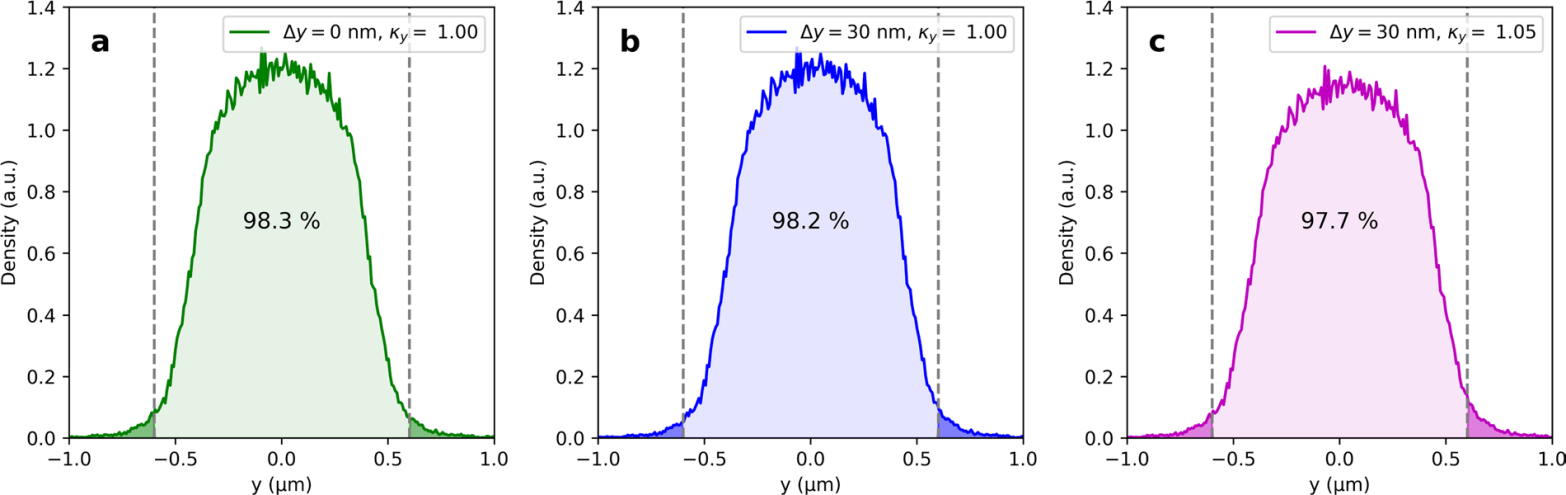
Transverse beam profiles for different combinations of position offset (Δ*y*) and beam size factor (${\kappa }_{y}$): (**a**) ideal case $\Delta y=0\,{\rm{nm}},{\kappa }_{y}=1.00$, (**b**) $\Delta y=30\,{\rm{nm}},{\kappa }_{y}=1.00$, (**c**) worst case $\Delta y=30\,{\rm{nm}},{\kappa }_{y}=1.05$). The aperture of the dielectric grating structure is shown in gray, dashed.

Good stability of beam position and size is required for the DLA modulation scheme, due to its micro-meter sized aperture. The electromagnetic field simulation for the proposed structure shows good homogeneity of the modulation strength (<0.05%) and negligible transverse forces across the channel gap. Therefore, beam position and size jitter will not affect the modulation strength but only the number of particles hitting the boundary of the dielectric grating. In the following, we estimate the fraction of particles being transmitted through the structure geometry defined above. An upper limit for the position jitter of the electron beam in the accelerator of SwissFEL is 10% of the beam size. The beam size measured along the machine typically shows jitter in the order of 3%. In the proposed strong focusing setup, energy jitter adds significantly to the beam size due to chromatic effects of the permanent magnet quadrupoles. Simulations using ELEGANT^23^ show an increase of the spot size by 1% for a typical energy error of 0.1%. For the calculation of the transmitted fraction we consider a maximum position offset of $\Delta y=30\,{\rm{nm}}$ and a beam size scaled by the factor ${\kappa }_{y}=1.05$. The beam profile distribution is shown in Fig. 8 for three different combinations: (a) $\Delta y=0\,{\rm{nm}},{\kappa }_{y}=1.00$, (b) $\Delta y=30\,{\rm{nm}},{\kappa }_{y}=1.00$, (c) $\Delta y=30\,{\rm{nm}},{\kappa }_{y}=1.05$. In the worst case scenario (c) a fraction of 2.3 of the electrons will scatter in the dielectric material of the structure. Towards high repetition rates above 100 Hz, radiation protection of the machine may become an issue. In this case, it might me required to increase the structure gap and sacrifice efficiency. The small loss of scattered particles in the tail of the electron beam do not reduce the stability of the FEL output power since the core of the beam is dominantly driving the SASE FEL process.

## Data Availability

The simulation data that support the findings of this study are available from the corresponding author upon reasonable request.
